# Relative extended haplotype homozygosity signals across breeds reveal dairy and beef specific signatures of selection

**DOI:** 10.1186/s12711-015-0113-9

**Published:** 2015-04-02

**Authors:** Lorenzo Bomba, Ezequiel L Nicolazzi, Marco Milanesi, Riccardo Negrini, Giordano Mancini, Filippo Biscarini, Alessandra Stella, Alessio Valentini, Paolo Ajmone-Marsan

**Affiliations:** Istituto di Zootecnica, UCSC, via Emilia Parmense 84, Piacenza, 29122 Italy; Fondazione Parco Tecnologico Padano, Via Einstein, Loc. Cascina Codazza, Lodi, 26900 Italy; Associazione Italiana Allevatori (AIA), Via Tomassetti 9, Rome, 00161 Italy; Center for Computational Chemistry and Cosmology, Scuola Normale Superiore, Via Consoli del Mare 2, Pisa, 56126 Italy; Istituto di biologia e biotecnologia Agraria (IBBA-CNR), Consiglio Nazionale delle Ricerche, Via Einstein, Cascina Codazza, Lodi, 26900 Italy; Dipartimento per l’Innovazione nei Sistemi Biologici, Agroalimentari e Forestali (DIBAF), via de Lellis, Viterbo, 01100 Italy

## Abstract

**Background:**

A number of methods are available to scan a genome for selection signatures by evaluating patterns of diversity within and between breeds. Among these, “extended haplotype homozygosity” (EHH) is a reliable approach to detect genome regions under recent selective pressure. The objective of this study was to use this approach to identify regions that are under recent positive selection and shared by the most representative Italian dairy and beef cattle breeds.

**Results:**

A total of 3220 animals from Italian Holstein (2179), Italian Brown (775), Simmental (493), Marchigiana (485) and Piedmontese (379) breeds were genotyped with the Illumina BovineSNP50 BeadChip v.1. After standard quality control procedures, genotypes were phased and core haplotypes were identified. The decay of linkage disequilibrium (LD) for each core haplotype was assessed by measuring the EHH. Since accurate estimates of local recombination rates were not available, relative EHH (rEHH) was calculated for each core haplotype. Genomic regions that carry frequent core haplotypes and with significant rEHH values were considered as candidates for recent positive selection. Candidate regions were aligned across to identify signals shared by dairy or beef cattle breeds. Overall, 82 and 87 common regions were detected among dairy and beef cattle breeds, respectively. Bioinformatic analysis identified 244 and 232 genes in these common genomic regions. Gene annotation and pathway analysis showed that these genes are involved in molecular functions that are biologically related to milk or meat production.

**Conclusions:**

Our results suggest that a multi-breed approach can lead to the identification of genomic signatures in breeds of cattle that are selected for the same production goal and thus to the localisation of genomic regions of interest in dairy and beef production.

**Electronic supplementary material:**

The online version of this article (doi:10.1186/s12711-015-0113-9) contains supplementary material, which is available to authorized users.

## Background

Advances in genomic technologies and the availability of single nucleotide polymorphism (SNP) markers have enabled genome-wide studies of the effect of selection in cattle [1,2]. Selection signals that result from environmental or anthropogenic pressures help us understand the processes that have led to breed formation. These studies are usually conducted with a “top-down” approach [3], from genotype to phenotype, whereby genomic data are statistically analysed to detect traces/marks/signs of directional selection. In analyses that aim at identifying selection signatures, the phenotype is considered in its broadest sense: breed, production aptitude or even adaptation to a specific environment. This approach holds the potential to investigate traits that are very expensive, difficult and sometimes impossible to study with classical GWAS (genome-wide association study) approaches, such as tolerance to extreme climates or various feeding and husbandry systems, resilience to diseases, etc. Therefore, results from these studies are complementary to those from GWAS for investigating the molecular mechanisms that underlie important biological processes [4]. Many methods have been proposed to scan for selection signatures at the genomic level [5] by analysing either within- or across-breeds patterns of diversity by comparing allele or haplotype frequencies and sizes, alleles that are segregating or fixed in populations, and to preferentially detect recent or ancient selection events [5-7]. Different methods have different sensitivities and robustness, *e.g.* they may be influenced to a different extent by marker ascertainment bias and uneven distribution of recombination hotspots along the genome.

Extended haplotype homozygosity (EHH), a method that identifies long-range haplotypes, was developed by Sabeti et al. [8] for applications in human genetics and has been applied to many animal species, including cattle [2,9,10]. Under a neutral evolution model, changes in allele frequencies are assumed to be driven only by genetic drift. In this scenario, a new variant will require many generations to reach a high frequency in the population, and the surrounding linkage disequilibrium (LD) will decay due to recombination events [11]. Conversely, in the case of positive selection, a rapid rise in frequency of a beneficial mutation in a relatively few generations will preserve the original haplotype structure (core haplotype), since the number of recombination events would be limited. Therefore, based on EHH, a positive selection signature is defined as a region characterized by strong and long-range LD and having an allele within an uncommonly high frequency haplotype.

The EHH method detects genomic regions that are candidates for having undergone recent selection and, unlike integrated haplotype score (iHS) [12], does not require the definition of ancestral alleles. In addition, it is suited to the analysis of SNP data, because it is less sensitive to ascertainment bias than other methods [4]. However, EHH is likely to generate a large number of false positive and false negative results, due to heterogeneous recombination rates along the genome [2]. An additional drawback that is shared by all selection signature methods, including EHH, is the challenge of robust inference, *e.g.* the ability to distinguish between true and spurious signals [13].

To partially account for these limitations, Sabeti et al. [8] developed the relative extended haplotype homozygosity (rEHH) method, which applies an empirical approach to assess the significance of signals. The rEHH of a core haplotype (*i.e.* short region in strong LD along the genome) is compared with the EHH value of other haplotypes at the same locus of the core haplotype, using these as a control for local variation in recombination rates. Therefore, it only identifies genomic regions, which carry variants under selection that are still segregating in the population. Although EHH and rEHH methods were developed for human population studies, they have been successfully applied to livestock species, such as pig [14] and cattle [2].

After domestication, which occurred about 10 000 years ago in the fertile crescent, taurine cattle colonized Europe and Africa and were selected to satisfy different human needs [15]. During the last century, anthropogenic pressure has led to the formation of hundreds of specialized breeds that are adapted to different environmental conditions and linked to local traditions, constituting a gene pool relevant for conservation [16]. Some of these breeds have experienced strong artificial selection for dairy, beef, or both production specializations [17]. The present study uses the rEHH method to identify signals of recent directional selection in dairy and beef production, using five Italian dairy, beef and dual purpose cattle breeds. We focused on significant core haplotypes that are shared by breeds selected for the same production type. Finally, we identified positional candidate genes within the genomic regions under selection and investigated their biological role.

## Methods

### Animals sampled and genotyping

A total of 4311 bulls from five Italian dairy, beef, and dual purpose breeds were genotyped with the Illumina BovineSNP50 BeadChip v.1 (Illumina, San Diego, CA), by combining genotyping efforts of two Italian projects (“SelMol” and “Prozoo”). The dataset included 101 replicates and 773 sire-son pairs, used for downstream quality checking of the data produced. The genotypes of 2954 dairy (2179 Italian Holstein and 775 Italian Brown), 864 beef (485 Marchigiana and 379 Piedmontese) and 493 dual purpose (Italian Simmental) bulls were available. Data quality control (QC) was performed in two steps: first on animals, independently in each breed, by applying the same filters and thresholds, and then on markers, across all individuals in the dataset. The first step excluded individuals with unexpectedly high (≥0.2%) Mendelian errors for father-son pairs and individuals with low call rates (≤95%). The second step excluded: (i) SNPs with more than 2.5% missing values in the whole dataset or completely missing in one breed; (ii) SNPs with a minor allele frequency less than 5%; and (iii) SNPs that were located on the sex chromosomes or for which chromosome assignment or physical position was lacking.

### Estimation of rEHH

Haplotypes were obtained by fastPHASE using the default options [18], and were run by breed and chromosome for each breed. Pedigree information for all bulls was provided by breed associations, and were used to filter out direct relatives (in father-son pairs, the son was maintained in the dataset and the father removed) and over-represented families (a maximum of five randomly chosen individuals per half-sib family was allowed). The final dataset containing these “less-related” animals is referred to as the “non-redundant” dataset and was used to calculate the within-breed pair-wise LD. The r^2^ statistic for all pairs of markers was calculated using PLINK v.1.0.7 [19]. The decay of LD was estimated by averaging r^2^ values as a function of marker distance, up to 1 Mb.

To test if population structure influenced rEHH detection, we repeated the whole Italian Holstein dataset analysis (*i.e.* the “redundant” dataset comprising father-son pairs and all available half-sibs per family) and focused on genes or gene clusters that are well known to be under recent selection in cattle (*i.e.* “control regions”). In particular, we focused on the *casein* gene cluster, the polled locus and on two coat colour genes (*MC1R* and *KIT* [2,13]).

EHH and rEHH were calculated by Sweep v.1.1 [8]. Some default program settings had to be modified to adapt the analysis to the bovine genome. Specifically, local recombination rates between SNPs were approximated to 1 cM per Mb. EHH and rEHH calculations were performed by breed and chromosome, using automatic haplotype core selection with default options, *i.e.* considering the longest non-overlapping haplotype cores and limiting haplotype cores to at least three and no more than 20 SNPs, as in Qanbari et al. [2]. To set an (empirical) rEHH significance threshold, we first split rEHH values into 20 bins with a frequency range of 5% each, then log-transformed within-bin values to achieve normality, and finally considered significant core haplotypes with a *p*-value less than 0.05. Although EHH and rEHH values were obtained for all core haplotypes, only those with a frequency greater than 25% were retained for further analyses.

### Breed grouping according to production type

Regions under putative selection for dairy and beef production were identified from significant core haplotypes that shared one or more SNPs in at least two breeds with the same production type. The dual purpose Italian Simmental was included in both dairy (Italian Holstein and Italian Brown) and beef breeds (Piedmontese and Marchigiana), since this breed potentially possesses haplotypes that have been selected for both production types. All downstream analyses were performed separately for the dairy and beef breeds.

### Detection and annotation of candidate genes

The genomic coordinates (in bp) of the regions shared by dairy or by beef breeds were used as inputs to retrieve gene information and annotation from the Biomart web interface (http://www.ensembl.org/biomart/martview). The resulting gene set was then used as input for a canonical pathway analysis by examining the functional relationships among the resulting genes using Ingenuity Pathway Analysis tool version 8.0 (IPA; Ingenuity® Systems, Inc, Redwood City, CA; http://www.ingenuity.com), coupled with a detailed examination of the literature. IPA operates with a proprietary knowledge database, providing pathway analysis for several species, including cattle. For IPA analysis, Fisher’s exact test following a Benjamini and Hochberg correction for multiple-testing was used to estimate the significance of each biological function.

## Results

### Quality control of the dataset

Reproducibility observed from analysis of 101 replicates in the whole dataset was greater than 99.8%. After the two quality control steps, 105 individuals and 9730 SNPs were removed. After phasing, 1292 additional individuals were removed to reduce the large number of sib-families present in the redundant dataset. The final dataset contained 44 271 SNPs and 1132, 514, 393, 410 and 364 individuals from Italian Holstein, Italian Brown, Italian Simmental, Marchigiana and Piedmontese breeds, respectively (Table 1).

**Table 1:** **Number of animals genotyped before and after quality control**

**Breed**	**Total genotyped**	**ED- 5% misAN**	**ED-REPL**	**ED- MEND**	**Cleaned**
**HOL**	2179	40	31	5	2093
**BRW**	775	6	16	4	749
**SIM**	493	6	6	2	479
**MAR**	485	37	38	-	410
**PIE**	379	5	10	-	364

Total number of animals genotyped and number of animals removed after quality control analysis; HOL = Holstein, BRW = Italian Brown, SIM = Simmental, MAR = Marchigiana, PIE = Piedmontese, ED- 5% misAN = number of animals excluded with call rates < 95%, ED-REPL = number of animals excluded because they are replicates, ED- MEND = number of animals excluded with Mendelian errors > 0.2%.

### Assessing the effect of population structure in control regions

**Figure 1:**
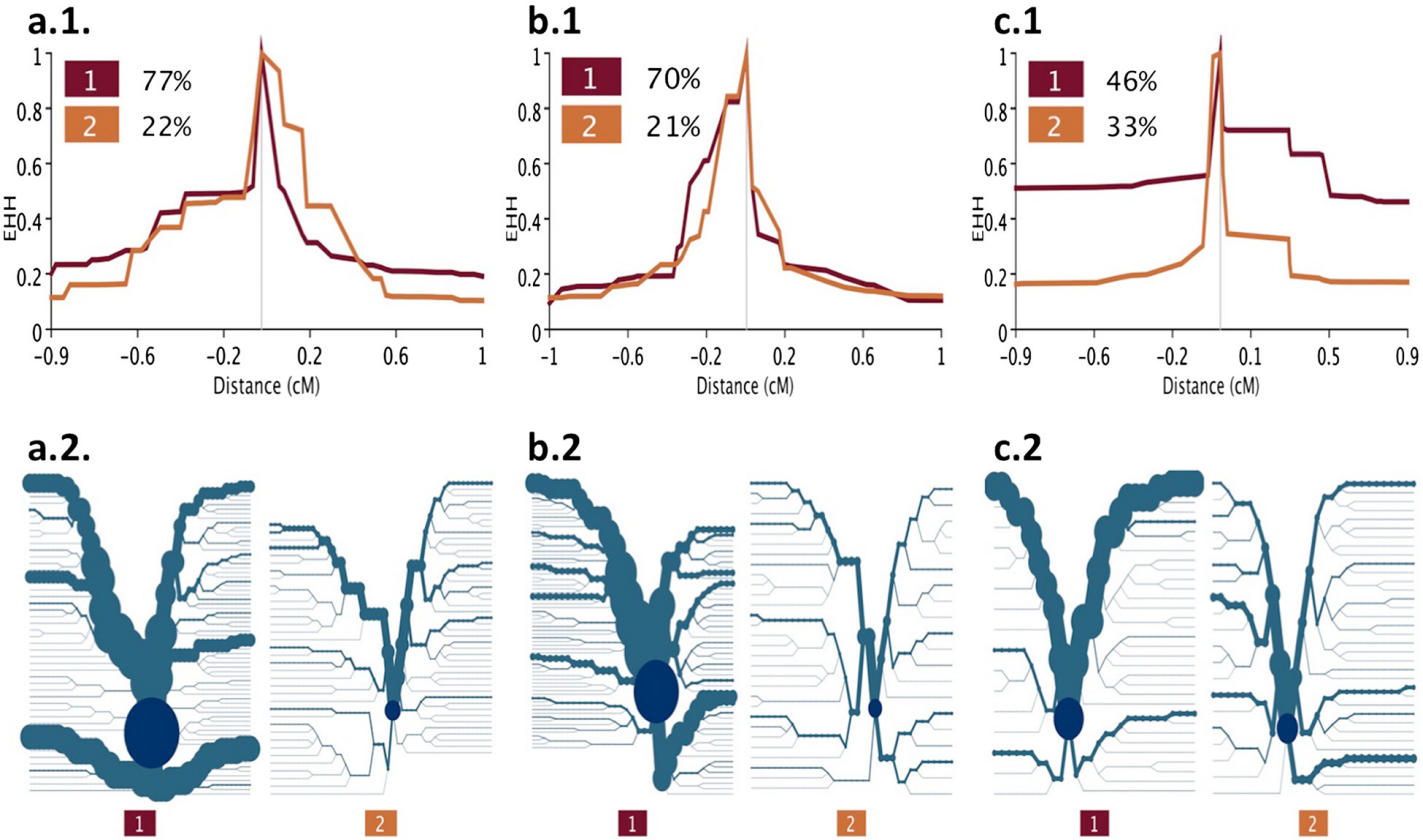
**EHH decay over distance (1) and bifurcation plots (2) in the Italian Holstein non-redundant dataset. (a.1)**, **(b.1)** and **(c.1)** show the decay of haplotype homozygosity as a function of distance for the two most frequent core haplotypes. **(a.2)**, **(b.2)** and **(c.2)** show haplotype bifurcation diagrams for the two most frequent core haplotypes at three control regions found to be significant rEHH in our study i.e. **(a)** polled locus, **(b)** *MC1R* gene and **(c)** *casein* gene cluster).

Comparison of rEHH at the four selected control regions (*casein* gene cluster, polled locus, *MC1R* and *KIT* genes) based on analysis of the redundant versus non-redundant datasets indicated that population structure (redundant vs. non-redundant datasets) has an influence on the detection of selection sweeps. In fact, in the redundant dataset, Sweep v1.1. detected only one significant core haplotype at the *casein* gene cluster, while in the non-redundant dataset significant haplotypes were also found at the polled locus and at the *MC1R* gene. No significant signal was detected at the *KIT* gene in either dataset (Table 2). Plots of EHH vs. distance for the two most frequent haplotypes around the genes with significant rEHH (*casein* gene cluster, polled locus and *MC1R*) are in Figure 1. All subsequent analyses were conducted on the non-redundant dataset which, although of smaller size, proved more informative than the entire dataset.

**Table 2:** **Comparison of rEHH signals in candidate regions in non-redundant and redundant dataset**

**Candidate region**	**BTA**	**Pos (bp)**	**Core haplotype range**	**Redundant**		**Non-redundant**	
				**CHFrq**	**rEHH -log(** ***P*** **) up/down** ^**1**^	**CHFrq**	**rEHH -log(** ***P*** **) up/down**
**Polled locus**	1	1981154	1897418-1981154	H1:0.79	0.93/0.37	H1:0.78	1.67*/1.74*
**MC1R**	18	13657912	13317720-14007505	H1:0.69	1.20/0.96	H1:0.69	0.77/1.67*
**KIT_ BOVIN**	6	72821175	72504921-72821175	H1:0.54	0.30/0.30	H1:0.54	0.33/0.48
**Casein cluster**	6	88427760	88350095-88452829	H1:0.47	0.94/1.39*	H1:0.46	1.48*/1.33*
	6	88427760	88350095-88452829	H2:0.32	0.02/0.03	H2:0.33	0.02/0.03

rEHH in candidate regions in both uncorrected (redundant) and corrected (non-redundant) datasets for population structure; BTA = *Bos taurus* autosome; CHFrq = core haplotype frequency; ^1^up-stream (left) and down-stream (right) of the core haplotype; *significant rEHH (p < 0.05).

### Detection of selection signatures

In total, 17 363, 17 801, 14 837, 13 814, and 12 747 core haplotypes with a frequency greater than 25% were detected in the Italian Holstein, Italian Brown, Italian Simmental, Marchigiana and Piedmontese breeds, respectively. The genome-wide distributions of *p* values for rEHH for each breed are in Additional file 1 [See Additional file 1: Figures S1, S2, S3, S4 and S5]. In total 838, 866, 740, 692 and 613 core haplotypes were found to be significant (p values ≤ 0.05) in the aforementioned breeds. Table 3 shows the distribution of total and significant core haplotypes per chromosome and breed.

**Table 3:** **Distribution of core haplotypes**

**BTA**	**Italian Holstein**		**Italian Brown**		**Italian Simmental**		**Marchigiana**		**Piedmontese**	
	**Hap** ^**2**^	**Sign.Hap** ^**3**^	**Hap** ^**2**^	**Sign.Hap** ^**3**^	**Hap** ^**2**^	**Sign.Hap** ^**3**^	**Hap** ^**2**^	**Sign.Hap** ^**3**^	**Hap** ^**2**^	**Sign.Hap** ^**3**^
**1**	1238	66	1228	68	1062	49	988	51	907	46
**2**	998	47	1040	52	830	44	822	42	735	33
**3**	913	48	1039	62	780	46	721	34	702	34
**4**	869	46	868	50	786	41	725	39	697	39
**5**	696	32	680	29	619	30	543	27	488	22
**6**	891	51	928	40	820	45	770	38	729	42
**7**	761	37	807	46	631	39	624	33	661	27
**8**	835	39	863	38	735	35	710	32	637	31
**9**	738	37	727	41	649	37	532	22	575	30
**10**	685	36	722	34	577	25	620	26	498	25
**11**	816	39	758	34	686	30	637	33	586	25
**12**	486	24	511	18	477	21	437	25	405	22
**13**	675	32	602	19	477	18	539	35	424	19
**14**	554	17	563	25	531	29	423	22	402	19
**15**	571	31	581	29	468	23	446	13	399	19
**16**	535	28	602	29	504	25	444	19	380	15
**17**	524	27	585	29	540	31	429	22	389	16
**18**	413	18	511	19	356	14	346	10	252	12
**19**	424	22	436	23	349	16	310	18	295	15
**20**	578	35	522	33	467	23	387	23	384	17
**21**	458	23	478	30	356	18	315	21	312	13
**22**	444	24	451	19	349	21	355	18	316	13
**23**	325	13	315	16	198	10	186	9	182	8
**24**	408	11	449	20	396	17	299	23	306	16
**25**	273	12	271	11	205	10	246	12	214	6
**26**	379	7	375	14	305	12	275	15	290	16
**27**	284	12	281	15	235	11	268	12	190	10
**28**	277	9	298	10	201	10	202	9	183	11
**29**	315	15	310	13	248	10	215	9	209	12
**TOT**	**17363**	**838**	**17801**	**866**	**14837**	**740**	**13814**	**692**	**12747**	**613**

Distribution of total and significant core haplotypes (up- and down-stream) per chromosome and breed; BTA = *Bos taurus* autosomes; ^2^number of detected core haplotypes with a frequency in the breed higher than 25%; ^3^number of significant core haplotypes at *p* ≤ 0.05.

### Comparison to previously reported data

A number of studies have searched for selection sweeps in Holstein [2], Brown [20] and Simmental [21] cattle. Since different methods are expected to identify different signatures, comparison with previous results is limited to those using the same method and breed(s) as in our study. There is currently only one study that reported rEHH results in (German) Holstein-Friesian cattle [2]. The number of core haplotypes found in our study in the candidate regions was lower than that in Qanbari et al. [2] (Table 4). For Holsteins, the two most significant candidate regions in both studies agreed (*casein* gene cluster and *somatostatin SST* gene), although it is impossible to determine if the haplotype under selection is the same, since this information was not provided in [2]. However, other genes considered significant in Qanbari et al. [2] (with *p*-values ranging from 0.04 to 0.10) were not significant in our study. When using the same loose significance threshold as in Qanbari et al. [2] (*p*-values ≤ 0.10), the *casein* gene cluster in this study was identified in Italian Brown (−log_10_*p*-value = 1.09) and the *SST* gene in Italian Simmental (−log_10_*p*-value = 1.26).

**Table 4:** **rEHH values in the candidate gene regions studied in** [2]

			**Holstein**			**Brown**			**Simmental**		
**Cand gene**	**BTA**	**Closest SNP (bp)**	**CH range**	**CH freq**	**rEHH-log(p)**	**CH range**	**CH freq**	**rEHH-log(p)**	**CH range**	**CH freq**	**rEHH-log(p)**
**DGAT1**	14	444 963	236 653–443 936	H1:0.57	-/0.11	443 936–763 332	H1:0.42	0.13/0.007	-	-	-
**Casein cluster**	6	88 391 612	88 350 098–88 452 835	H1:0.46	1.48*/1.33*	88 326 012–88 452 835	H2:0.68	0.80/1.09	88 350 098–88 452 835	H1:0.44	0.37/0.22
			88 350 098–88 452 835	H2:0.33	0.18/0.30	-	-	-	-	-	-
			-	-	-	-	-	-	88 350 098–88 452 835	H3:0.34	0.22/0.45
**GH**	19	49 652 377	-	-	-	-	-	-	-	-	-
**GHR**	20	33 908 597	-	-	-	-	-	-	-	-	-
**SST**	1	81 376 956	81 283 585–81 376 961	H1:0.36	2.00**/1.89**	-	-	-	81 318 451–81 376 961	H1:0.42	1.26/0.53
			81 283 585–81 376 961	H2:0.29	0.063/0.084	-	-	-	81 318 451–81 376 961	H2:0.42	0.06/0.27
**IGF-1**	5	71 169 823	-	-	-	-	-	-	-	-	-
**ABCG2**	6	37 374 911	-	-	-	37 317 020–38 256 889	H1:0.44	0.31/0.27	-	-	-
			-	-	-	37 317 020–38 256 889	H1:0.40	0.29/0.31	-	-	-
**Leptin**	4	95 715 500	-	-	-	-	-	-	-	-	-
**LPR**	3	85 569 203	85 497 108–85 594 551	H1:0.47	0.91/0.72	85 497 108–85 794 693	H1:0.68	0.80/0.63	85 497 108–85 794 693	H1:0.63	0.02/0.05
			85 497 108–85 594 551	H2:0.41	0.27/0.25	-	-	-	-	-	-
**PIT-1**	1	35 756 434	-	-	-	-	-	-	-	-	-

Cand gene = candidate gene; BTA = *Bos taurus* autosomes; CH = core haplotype; freq = frequency. *0.05, **0.01.

### Shared signatures between breeds

Significant core haplotypes were aligned across breeds to identify those that were shared by dairy or beef breeds. Since breeds can be considered as independent sets of observations, shared signatures are more likely to represent real effects rather than false positives. A total of 123 significant core haplotypes (2.2% of the genome), with an average length 216 932 bp, were shared by at least two dairy breeds [See Additional file 2: Table S1]. For beef breeds, 142 core haplotypes (1.7% of the genome) were shared by at least two breeds, with an average length of 190 994 bp. Only 82 and 87 of the shared core haplotypes for dairy and beef breeds, respectively, contained genes. These were considered as positional candidate genes under positive selection and were further investigated.

### Gene set annotation and pathway analysis

**Figure 2:**
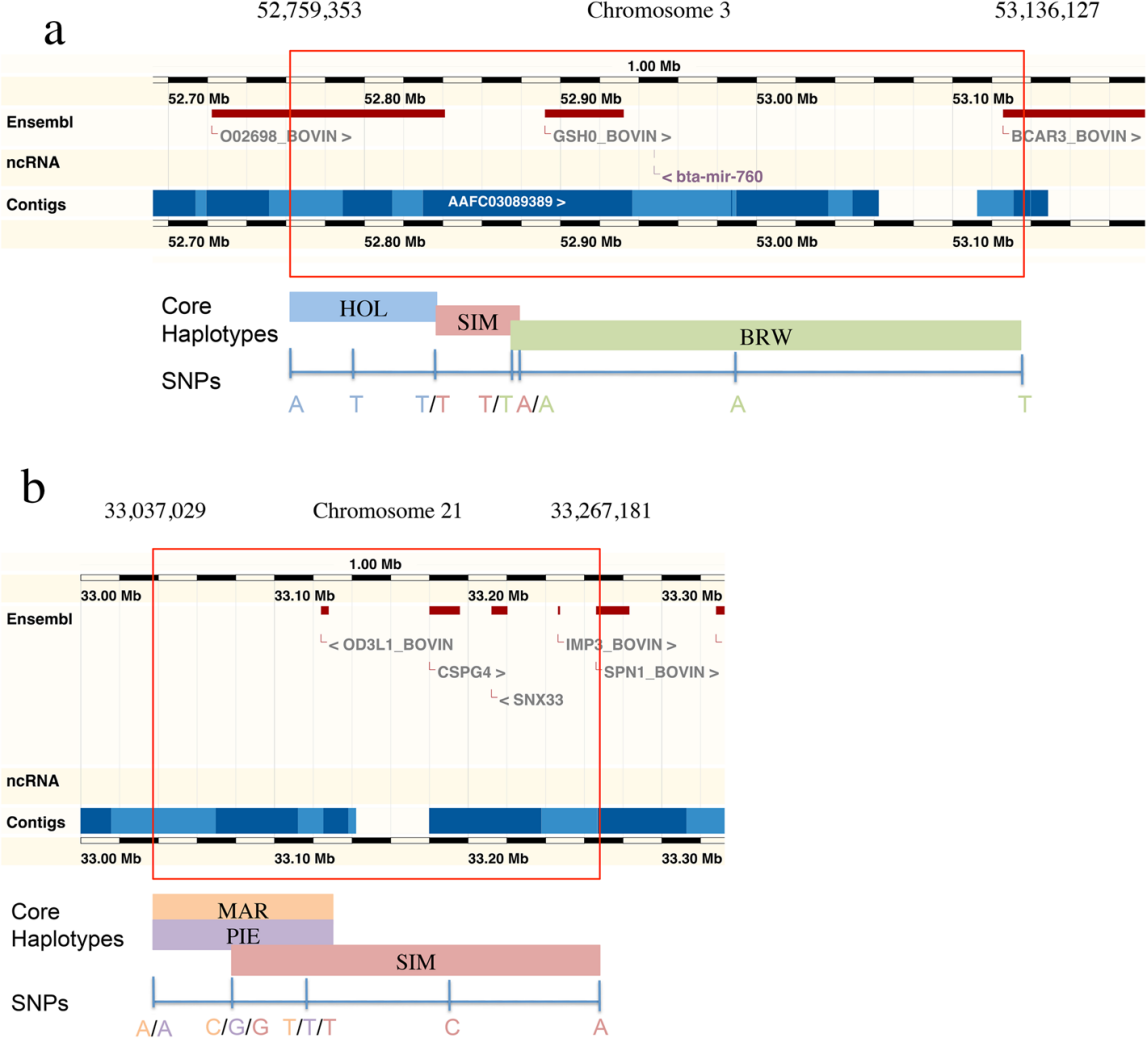
**Genomic location of the selection signatures shared among the studied breeds. (a)** Genes in Ensembl tracks are displayed as red boxes; core haplotypes and SNPs are coloured in orange (Marchigiana; MAR), in purple (Piedmontese; PIE) and pink (Simmental; SIM). **(b)** Genes in Ensembl tracks are displayed as red boxes; core haplotypes and SNPs are coloured in blue (Holstein; HOL), in green (Italian Brown; BRW) and pink (Simmental; SIM).

A total of 244 and 232 annotated genes fell within the regions under selection in dairy and beef breeds, respectively (Table 5 and [See Additional file 2: Table S1]). Among these, eight genes were shared by all three dairy breeds and 11 by all three beef breeds (see Figure 2 as an example).

**Table 5:** **Statistics on common significant core haplotypes in dairy and beef breeds**

	**Dairy breeds**				**Beef breeds**			
**BTA** ^**1**^	**Sign.CH** ^**2**^	**Nb genes** ^**3**^	**Sum CH size** ^**4**^	**Avg. CH size** ^**5**^	**Sign.CH** ^**2**^	**Nb genes** ^**3**^	**Sum CH size** ^**4**^	**Avg. CH size** ^**5**^
**1**	7	11	1521608	138328	7	13	2263213	174093
**2**	5	9	2216685	246298	8	11	2658549	241686
**3**	2	5	1619336	323867	7	21	3755847	178850
**4**	7	21	5956755	283655	6	11	1761288	160117
**5**	4	17	4506119	265066	5	11	1251127	113739
**6**	2	4	1467738	366934	5	12	5149692	429141
**7**	6	30	4842969	161432	3	28	11889191	424614
**8**	0	0	0	0	4	12	3512215	292685
**9**	4	7	1554863	222123	2	6	1106768	184461
**10**	4	17	8839762	519986	2	3	573138	191046
**11**	6	8	1841802	230225	1	1	100439	100439
**12**	2	8	4244133	530517	1	1	536461	536461
**13**	3	8	1933026	241628	2	8	985376	123172
**14**	0	0	0	0	3	9	692397	76933
**15**	5	9	1415405	157267	2	2	189094	94547
**16**	3	6	1302506	217084	4	6	905719	150953
**17**	3	4	471046	117762	2	9	1551010	172334
**18**	0	0	0	0	3	23	2961718	128770
**19**	3	23	5744143	249745	2	2	116938	58469
**20**	1	2	148886	74443	2	4	627971	156993
**21**	3	7	1930206	275744	1	5	1150760	230152
**22**	3	5	620674	124135	3	10	2411751	241175
**23**	0	0	0	0	1	1	68421	68421
**24**	2	18	9166004	509222	2	4	803770	200942
**25**	2	11	2016602	183327	1	3	329199	109733
**26**	2	4	466134	116534	4	7	776080	110869
**27**	1	1	631792	631792	2	3	1004717	334906
**28**	0	0	0	0	1	1	93464	93464
**29**	2	9	935209	103912	1	5	798300	159660
**TOT**	82	244	65393403	216932	87	232	50024613	190994

^1^ *Bos taurus* autosomes; ^2^number of significant core haplotypes (*P <* 0.05); ^3^number of genes identified in the significant regions; ^4^sum of significant core haplotypes size, in bp; ^5^average size of significant core haplotypes, in bp.

All identified genes were submitted to pathway analyses. The most interesting genes for dairy breeds were *breast cancer anti-estrogen resistance 3* (*BARC3*) and *pituitary glutaminyl cyclase* (*QPCT*), which are directly connected with the metabolism of the mammary gland [22,23]. *Solute carrier family 2, member 5* (*SLC2A5*) facilitates glucose/fructose transport [24], and *zeta-chain (TCR) associated protein kinase 70 kDa* (*ZAP70*) plays a critical role in T-cell signalling [25]. Calpain is another important complex that, together with *calpain-3* (*CAPN3),* mediates epithelial-cell death during mammary gland involution [26]. Furthermore, RAS guanyl nucleotide-releasing protein (RASGRP1) activates the Erk/MAP kinase cascade, regulates the development of T- and B-cells, homeostasis and differentiation, and is involved in regulation of breast cancer cells [27-29].

*Chondroitin sulfate proteoglycan 4* (*CSPG4*) and *snurportin-1* (*SNUPN*) are the most interesting genes that were shared among all beef cattle breeds investigated. *CSPG4* is related to meat tenderness, while *SNUPN* is an imprinted gene that has an important role in embryo development and is involved in human muscle atrophy [30].

**Figure 3:**
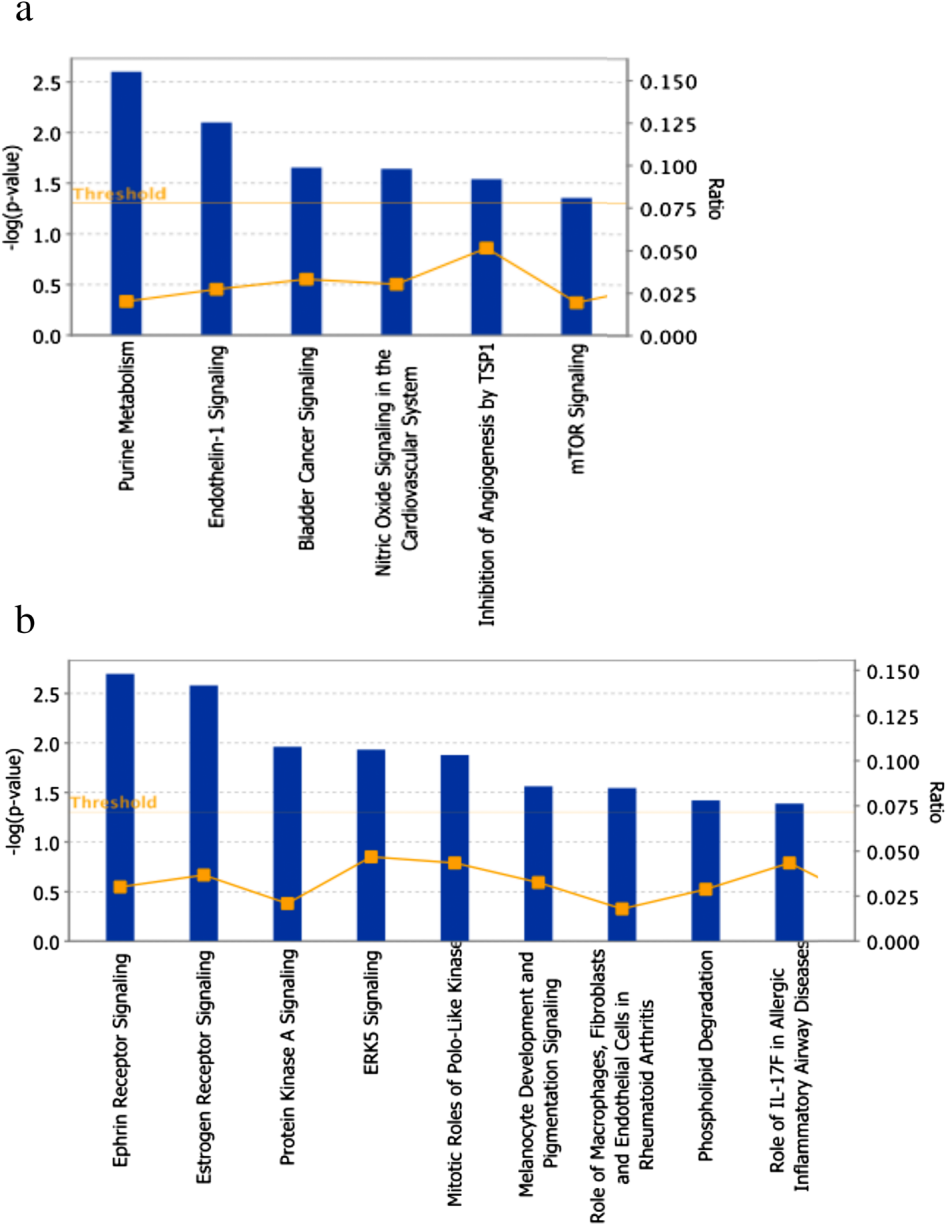
**Bar plot of statistically significant canonical pathways.** *P*-values were corrected for multiple-testing using the Benjamini-Hochberg method and are presented in the graph as -log(*p*-value). The bar represents the percentage of genes in a given pathway that meet the cut-off criteria within the total number of molecules that belong to the function. **(a)** Bar plot of statistically significant canonical pathways in dairy cattle breeds. **(b)** Bar plot of statistically significant canonical pathways in beef cattle breeds.

A total of six and nine statistically significant canonical pathways (FDR ≤ 0.05; −log_10_(FDR) ≥ 1.3) were identified using IPA for dairy and beef breeds, respectively (Figure 3 and [See Additional file 3: Table S2]). For the dairy breeds, the most significant canonical pathway was identified for purine metabolism (−log_10_(FDR) = 2.6), which supports the highly synthetic processes in the mammary epithelium [See Additional file 4: Figure S6]. In beef breeds, the signal for *ephrin receptor* (−log10(FDR) = 2.7) was the most significant canonical pathway [See Additional file 5: Figure S7]. Among other functions, *ephrin receptor* is known to promote muscle progenitor cell migration before mitotic activation [31]. All other canonical pathways are reported in Table S2 [See Additional file 3: Table S2].

## Discussion

In this study, the genotypes of more than 4000 bulls from five Italian breeds were analysed for putative dairy and beef selection signatures. Strict data quality control was applied to reduce possible sources of bias from genotyping errors and population structure. In particular, the confounding effect of population structure was investigated by replicating part of the analyses without excluding a large number of close relatives and without balancing family members in the dataset. Assessment of the effect of population structure on rEHH results was restricted to four control regions that are known to be under selection in Italian Holstein, namely the *casein* gene cluster, the polled locus, and the *MC1R* and *KIT* genes. This breed was selected for two reasons: (i) according to our data it is a highly structured breed and (ii) it allowed comparing our results with a previous study [2]. Although analyses conducted on both redundant and non-redundant datasets identified rEHH signals in these regions, the non-redundant dataset produced five significant rEHH signals, compared to only one in the redundant dataset (Table 2). These results highlight the confounding effect of the presence of close relatives in the dataset and, consequently, the improved ability to detect a significant selection signature when correcting for population structure.

Due to pedigree links, population stratification rather than selection leads to an over-representation of haplotypes that are present in large families (*e.g.* sires that pass half of their genetic material to their sons). For this reason, for the full analyses, all sire-son pairs were removed after haplotype phasing (retaining only the sons), and half-sib families were restricted to a maximum of five randomly chosen individuals, to reduce family over-representation. This threshold was a compromise between limiting haplotype redundancy and retaining sufficient information to detect signals; reducing half-sib families to only one individual (which would have been the most rigorous choice), would have led to an excessive reduction of the dataset. The progeny-tested Italian bulls analysed in this study are highly related, especially those of the dairy type, and if the most stringent threshold had been applied, 82% of the Italian Holstein individuals would have been removed.

The three significant control regions (Table 2) in the non-redundant dataset showed a slightly different EHH decay over distance, as shown in the bifurcation plots of Figure 1. Here, EHH values are reported, since they are graphically easier to interpret. The two most frequent haplotypes for the polled locus showed a similar EHH pattern: high values (i.e. ~1) close to the core haplotype and a rapid decay to 0.2 at ~1 cM down- and up-stream (Figure 1.a). The second haplotype, however, was excluded from this analysis, since its frequency was lower than the threshold that was set (<25%). Interestingly, the cumulative frequency of these two haplotypes in the whole Italian Holstein population was 99%, e.g. nearly all individuals carried these two (core) haplotypes. A similar pattern was observed for the two most frequent haplotypes in the *MC1R* gene (Figure 1b). In contrast to the polled locus, high EHH values (e.g. > 0.5) were maintained at distances of more than 200 kb up- and down-stream from the core haplotype for the *MC1R* gene, which potentially indicates more recent and strong selection. A more conserved haplotype was particularly evident for the *casein* gene cluster (Figure 1c), with EHH values greater than 0.6 at distances of more than ~1 Mb up- and down-stream from the core haplotype. Interestingly, similar values (both in terms of haplotype frequency and EHH) were reported in [2].

We also compared results of all candidate regions investigated in [2], our results only partially overlapped with those reported by Qanbari et al. [2]. Common signals were found at the *casein* gene cluster (see above) and the SST gene, while Qanbari et al. [2] found significant signals also in other regions. These inconsistencies may be due to the presence of different sires in the analyses, different dataset sizes, or to the close relative reduction procedure that we adopted to decrease the effect of population structure and consequent bias. However, poor agreement across studies is similarly observed in human studies and is often due to: (i) use of different within- and between-populations statistics that potentially identify selection signatures with different characteristics (ancient/recent, segregating/fixed, under directional/balancing selection), (ii) high rate of false positive/negative results, and (iii) different ways of accounting for population structure and background selection [32]. In a recent study, Mancini et al. [33] estimated the fixation index (Fst) in the same populations here investigated and identified signals that do not overlap with those reported here. Although at least a partial overlap was expected, this could be explained by the intrinsic differences between Fst and rEHH methods. By comparing two populations (or groups of populations), Fst is much more efficient in capturing large allele frequency differences between breeds and thus identifies “outlier” SNPs that are fixed or close to fixation for opposite alleles. This means that the identified signals are usually markers that have been differentially selected for a relatively large number of generations (e.g. “old” selection). Conversely, the rEHH identifies long haplotypes that segregate at high frequency in the population, and thus are, by definition, recent.

**Figure 4:**
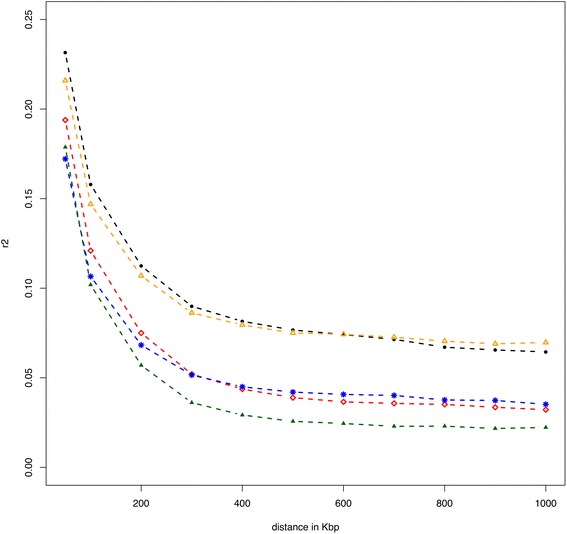
**Multi-breed average linkage disequilibrium against physical distance (in kb).** Marchigiana (blue stars), Piedmontese (green filled triangles) and Italian Simmental (red diamonds) breeds show a lower persistence of LD over distance than Italian Holstein (black filled circles) and Italian Brown (orange triangles) breeds.

The number of total and significant core haplotypes identified by the Sweep software was highest for the Italian Brown and lowest for the Piedmontese breed. Since rEHH methods rely heavily on population LD, the average LD at different genomic distances was estimated for each breed (Figure 4). Although values of LD based on sequence data decay at shorter distances than the values presented here [34], this analysis highlighted a general positive correlation between the level of LD over distance and the number of total and significant core haplotypes found. However, considering that rEHH is a relative measure, the larger number of significant core haplotypes identified for dairy breeds was likely due to the higher selective pressure (and thus a higher local LD at specific loci) in dairy compared to beef breeds.

Significance tests used to detect selection signatures should measure the probability of a statistic being an outlier value compared to its expected distribution under a neutral model. However, no reliable neutral model has so far been developed for cattle because of the complexity of the demographic history of this species [13]. As a consequence, empirical rather than model-based significance tests are generally used to detect selection signatures. Accordingly, we considered outlier values as those falling in the 5% plus variant tail of the rEHH distribution. We kept the within-breed significance threshold loose without correcting it for multiple-testing, but considered only signals shared by two or more independent breeds of the same production type.

The parallel comparison of results from independent analyses of different breeds allowed us to reach a double objective: (i) identification of putative regions under (recent) selection in breeds with different production purposes, which was the main objective of this study, and (ii) reduction of the rate of false positives, since multi-breed analyses served as internal controls. Since the rEHH method does not consider phenotypic information, a significant signal might arise because: (i) the core haplotype is actually under selective pressure or (ii) the result is a false positive, *i.e.* caused by chance, population structure or any other driving force. However, even considering an unrealistic scenario with no false positives, a proportion of all signals will actually be selection signatures due to selection pressure on traits other than those specific to dairy or beef traits. This is because only a few dairy and beef breeds were analysed and beef breeds share a number of traits that are not directly related to dairy or beef production, such as coat colour, polled/horned, etc. Even considering this limitation, to our knowledge, this is the first multi-breed study in dairy and beef cattle that applies such a strategy to reduce the rate of false positives, at the cost of a possible loss of information due to higher false negative rates. Significant signals shared by dairy and beef breeds were used for downstream gene annotation and pathway analyses on positional candidate genes to investigate the biological processes behind the genomic signals. Only the most significant pathways for dairy and beef breeds will be discussed in detail in the following.

### Dairy breeds

Putative signals of selection were found in regions that contain the *BARC3* and *QPCT* genes, and these were shared among all three dairy breeds. To date, neither of these genes have been studied in cattle. However, human studies have shown that these genes are linked to mammary gland metobolism and calcium regulation. *BARC3* is involved in integrin-mediated cell adhesion and signaling, which is required for mammary gland development and function [23]. *QPCT* is associated with low radial body mineral density (BMD) in adult women [22]. Another interesting candidate gene is *SLC2A5*, which acts as a fructose transporter in the intestine and has a significant role in energy balance of dairy cows [35]. The detection of this gene in a dairy cattle specific candidate region is surprising, since, in theory, there should be little need for transporting glucose and/or fructose in the ruminant intestinal tract because simple carbohydrates are degraded into volatile fatty acids (VFA) in the rumen [36]. However, it is known that large amounts of starch bypass the rumen in cows fed on diets that are rich in cereal grains [24]. This bypassed starch needs to be digested in the small intestine and then absorbed, to avoid high levels of glucose in the large intestine.

We detected candidate regions that contained the *calpain complex* and *calpain-3* (*CAPN3*) genes in the dairy breeds, as reported by Utsunomiya et al. [7]. Although calpain is known to be involved in postmortem meat tenderization, it is also related to dairy metabolism, since muscle breakdown promoted by *calpain* provides an energy source for milk production especially at the beginning of lactation [37]. In addition, as reported by Wilde et al. [26], the *calpain-calpastatin* system is related to the programmed cell death of alveolar secretory epithelial cells during lactation. The *Zap70* gene encodes a cytoplasmic protein tyrosine kinase that is related to the immune system and plays a central role in T-cell responses, as a component of the T-cell receptor [38]. Bonnefont et al. [25] reported that the *Zap70* gene was up-regulated in somatic cells present in the milk of sheep infected by *Staphylococcus aureus* and *Staphylococcus epidermidis*, which suggests an association with mastitis resistance.

Purine metabolism was the most significant canonical pathway in dairy breeds [See Additional file 4: Figure S6]. In a gene expression analysis on human breast milk fat globules, Maningat et al. [39] identified purine metabolism as the most significant pathway. Synthesis and breakdown of purine is essential in the tissue metabolism of many organisms, and in particular in that of the mammary gland during lactation.

Another interesting canonical pathway was endothelin signalling (Figure 3). Endothelin functions as a vasoconstrictor and is secreted by endothelial cells [40]. Acosta et al. [41] reported that in cattle, endothelins are involved in the follicular production of prostaglandins and the regulation of steroidogenesis in the mature follicle. In a recent study, Puglisi et al. [42] confirmed that endothelins, in particular *EDNRA* (a potential biomarker for fertility in cow) and *endothelin convertin enzyme 1*, are involved in a reproductive disorder in cows.

### Beef breeds

A suggestive signature of selection in all three beef breeds was found in the region of the *CSPG4* gene, which belongs to the *chondroitin sulfate proteoglycan* (*CSPG*) gene family. CSPG are proteoglycans that consist of a protein core and a chondroitin sulfate side chain. They are known to be structural components of a variety of tissues, including muscle, and to play key roles in neural development and glial scar formation. They are involved in cellular processes, such as cell adhesion, cell growth, receptor binding, cell migration, and interactions with other extracellular matrix constituents.

Many studies have reported the role of proteoglycans in the determination of meat texture of several bovine muscles [43]. Dubost et al. [44] highlighted a direct role of proteoglycans in cooked meat juiciness. Another putative signal of selection was found on the *RB1-inducible coiled-coil 1* (*RB1CC1*) gene. This gene plays a crucial role in muscular differentiation and its activation is essential for myogenic differentiation [45]. The *monoacylglycerol acyltransferase* (*MGAT3*) gene catalyses the synthesis of diacylglycerol (DAG) using 2-monoacylglycerol and fatty acyl coenzyme A. This enzymatic reaction is fundamental for the absorption of dietary fat in the small intestine. In a study on five Chinese cattle breeds, Sun et al. [46] reported that the *MGAT3* gene is associated with growth traits. The *cold inducible RNA binding protein* (*CIRPB*) gene may be part of a compensatory mechanism in muscles that undergo atrophy. It preserves muscle tissue mass during cold-shock responses, aging and disease [47]. *SNUPN* is an imprinted gene that is expressed monoallelically, depending on its parental origin. *SNUPN* plays important roles in embryo survival and postnatal growth regulation [48,49]. Ephrin receptor signalling was the top canonical pathway identified by IPA and has interesting biological roles for meat production [See Additional file 5: Figure S7]. Indeed, this pathway is important for muscle tissue growth and regeneration by participating in the correct positioning and formation of the neural muscular junction [31].

## Conclusions

In this study, we analysed candidate selection signatures at the genome-wide level in five Italian cattle breeds. Then, we used a multi-breed approach to identify the genomic regions shared among cattle breeds selected for dairy or beef production. This approach increased the potential of pin-pointing regions of the genome that play important roles in economically relevant traits. Moreover, gene annotation and pathway analyses were used to describe the gene functions in the regions potentially under recent positive selection.

Specifically, dairy cattle genes that are likely to be under directional selection are related to feeding adaptation (increasing levels of starch in the diet), mammary gland metabolism and resistance to mastitis, while putative regions under selection in beef cattle are related to animal growth, meat texture and juiciness. Considering that annotation for the bovine genome is not as accurate as for the human genome, the biological interpretation of selection signatures can be derived based only on genes that are located near candidate regions. Moreover, novel information in humans suggests that many selected variations are not located within genes and coding regions, but in regulatory sites that have been identified within the ENCODE project [32]. These may control the expression of entire genomic regions or genes located at a relevant distance from the selected site, making biological interpretation more complex.

Future studies using denser SNP chips or whole-genome sequencing that provide information not subjected to ascertainment bias [34], may increase the resolution of our analysis and, together with increasing knowledge on the control of gene expression, should validate our results.

## Additional files

Additional file 1: Figures S1, S2, S3, S4 and S5. Genome-wide map of P-values for core haplotypes for Italian Holstein, Italian Brown, Italian Simmental, Marchigiana and Piedmontese breeds, respectively. The file is a .zip compressed document including five .tiff images i.e. Figures S1, S2, S3, S4 and S5 that show the genome-wide map of P-values for core haplotypes with a frequency higher than 0.25 for Italian Holstein (HOL), Italian Brown (BRW), Italian Simmental (SIM), Marchigiana (MAR) and Piedmontese (PIE) breeds, respectively. Dashed lines represent the cut-off level of 0.01. Additional file 2: Table S1. Significant core haplotypes and genes shared between dairy and beef cattle breeds. Significant core haplotypes (*p*-value ≤ 0.05; haplotype frequency ≥ 0.25) shared among dairy and beef cattle breeds and the relative genes that intersect with the core haplotypes. The file is a .xls document that includes four sheets. The first two sheets show the significant core haplotypes for dairy and beef cattle breeds and the other two sheets show genes intersecting with the significant core haplotypes for dairy and beef cattle breeds. Additional file 3: Table S2. Ranking of canonical pathways in dairy or beef cattle breeds. Ranking of canonical pathways in dairy or beef cattle breeds with the list of corresponding gene symbols, ratio and –log10 of the *p*-values for each canonical pathway. Additional file 4: Figure S6. Genes detected under recent positive selection in dairy cattle and involved in the purine metabolism canonical pathway. Description: In Figure S6, nodes in red correspond to genes identified in core haplotypes that overlap in all three breeds of each production type, whereas those in green depict overlapping core haplotypes in at least two of those breeds. Additional file 5: Figure S7. Genes detected under recent positive selection in beef cattle and involved in the ephrin metabolism canonical pathway. In Figure S7, nodes in red correspond to genes identified in core haplotypes that overlap in all three breeds of each production type, whereas those in green depict overlapping core haplotypes in at least two of those breeds.
