# Supplementary figures and images for: Relative extended haplotype homozygosity signals across breeds reveal dairy and beef specific signatures of selection
Source: Genet Sel Evol. 2015 Apr 2;47(1):25. doi: 10.1186/s12711-015-0113-9 (PMC4383072; doi:10.1186/s12711-015-0113-9)

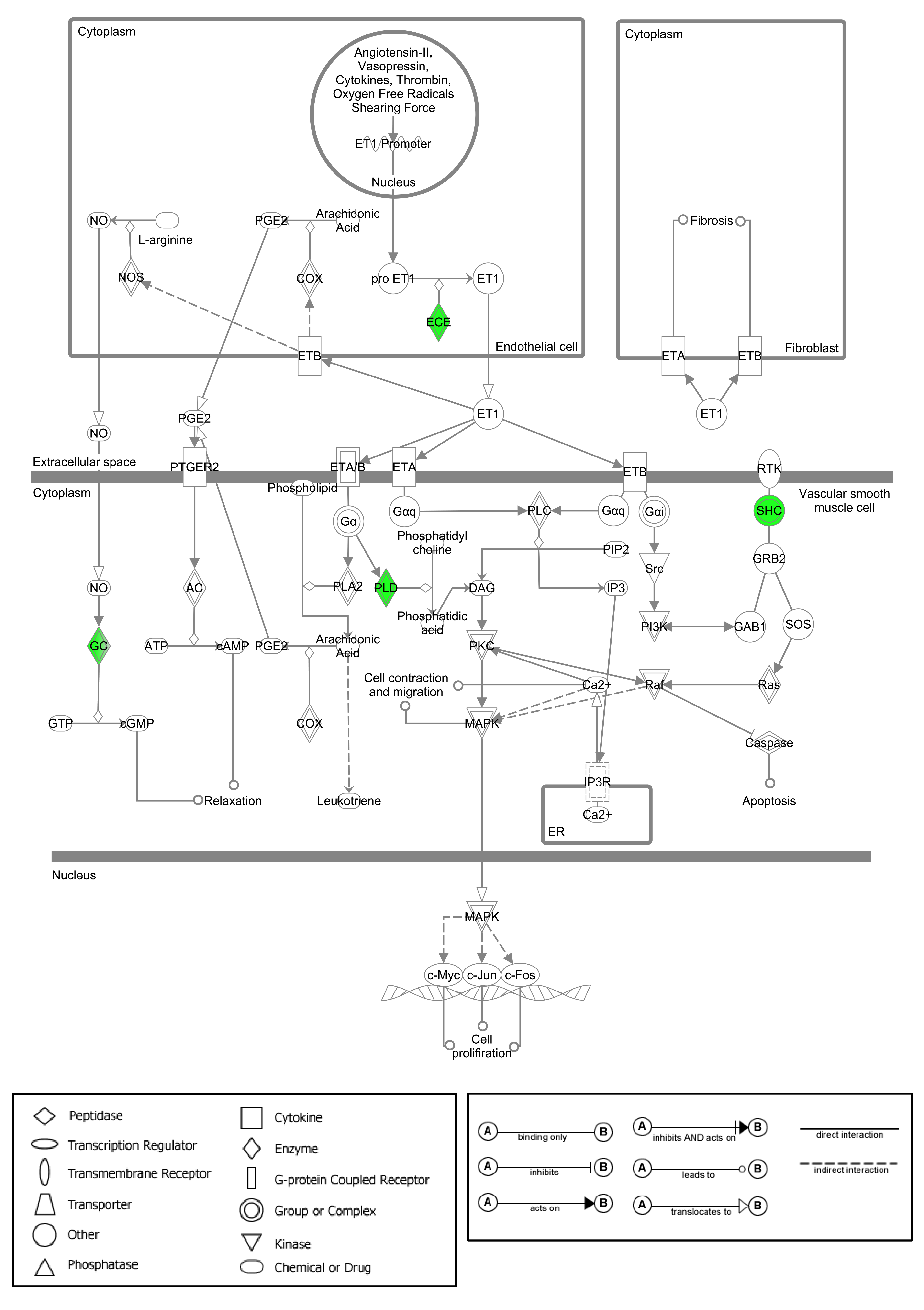

Supplement: Additional file 3: Table S2. — Ranking of canonical pathways in dairy or beef cattle breeds. Ranking of canonical pathways in dairy or beef cattle breeds with the list of corresponding gene symbols, ratio and –log10 of the p-values for each canonical pathway. [file 12711_2015_113_MOESM3_ESM.tiff]

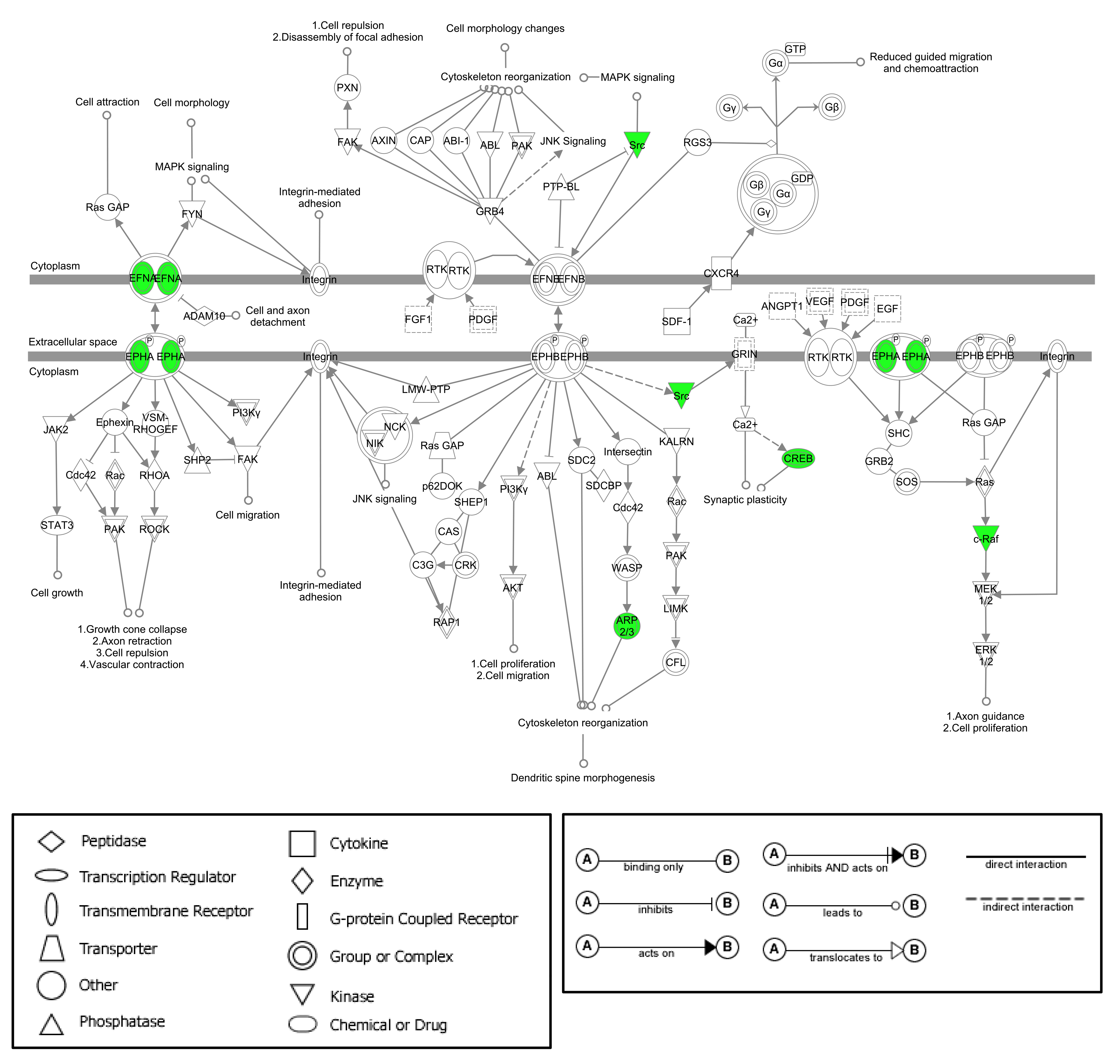

Supplement: Additional file 4: Figure S6. — Genes detected under recent positive selection in dairy cattle and involved in the purine metabolism canonical pathway. Description: In Figure S6, nodes in red correspond to genes identified in core haplotypes that overlap in all three breeds of each production type, whereas those in green depict overlapping core haplotypes in at least two of those breeds. [file 12711_2015_113_MOESM4_ESM.tiff]
